# Treatment adherence with the easypod™ growth hormone electronic auto-injector and patient acceptance: survey results from 824 children and their parents

**DOI:** 10.1186/1472-6823-11-4

**Published:** 2011-02-04

**Authors:** Mauro Bozzola, Michel Colle, Maria Halldin-Stenlid, Sylvain Larroque, Monia Zignani

**Affiliations:** 1 Paediatric Department, University of Pavia, IRCCS San Matteo Foundation, Pavia, Italy; 2 25 rue Boudet, Bordeaux, France; 3 Department of Women's and Children's Health, Uppsala University, Uppsala, Sweden; 4 Merck Serono S.A. - Geneva, Switzerland, an affiliate of Merck KGaA, Darmstadt, Germany

## Abstract

**Background:**

Accurately monitoring adherence to treatment with recombinant human growth hormone (r-hGH) enables appropriate intervention in cases of poor adherence. The electronic r-hGH auto-injector, easypod™, automatically records the patient's adherence to treatment. This study evaluated adherence to treatment of children who started using the auto-injector and assessed opinions about the device.

**Methods:**

A multicentre, multinational, observational 3-month survey in which children received r-hGH as part of their normal care. Physicians reviewed the recorded dose history and children (with or without parental assistance) completed a questionnaire-based survey. Children missing ≤2 injections per month (92% of injections given) were considered adherent to treatment. Adherence was compared between GH treatment-naïve and treatment-experienced children.

**Results:**

Of 834 recruited participants, 824 were evaluated. The median (range) age was 11 (1-18) years. From the recorded dose history, 87.5% of children were adherent to treatment over the 3-month period. Recorded adherence was higher in treatment-naïve (89.7%, n = 445/496) than in treatment-experienced children (81.7%, n = 152/186) [Fisher's exact test FI(X) = 7.577; *p *= 0.0062]. According to self-reported data, 90.2% (607/673) of children were adherent over 3 months; 51.5% (421/817) missed ≥1 injection over this period (mainly due to forgetfulness). Concordance between reported and recorded adherence was 84.3%, with a trend towards self-reported adherence being higher than recorded adherence. Most children liked the auto-injector: over 80% gave the top two responses from five options for ease of use (720/779), speed (684/805) and comfort (716/804). Although 38.5% (300/780) of children reported pain on injection, over half of children (210/363) considered the pain to be less or much less than expected. Given the choice, 91.8% (732/797) of children/parents would continue using the device.

**Conclusions:**

easypod™ provides an accurate method of monitoring adherence to treatment with r-hGH. In children who received treatment with r-hGH using easypod™, short-term adherence is good, and significantly higher in treatment-naïve children compared with experienced children. Children/parents rate the device highly. The high level of acceptability of the device is reflected by a desire to continue using it by over 90% of the children in the survey.

## Background

Recombinant human growth hormone (r-hGH) is used to treat growth hormone deficiency (GHD) in adults and short stature in children [1-3]. Treatment improves growth in children whose short stature is caused by GHD, or in those for whom short stature is associated with conditions such as Turner syndrome (TS), chronic renal failure (CRF) or being born small for gestational age (SGA) [1]. Early intervention with long-term r-hGH treatment improves adult stature, with some patients reaching target final height. However, lack of adherence hampers growth potential [4,5].

Maintaining commitment to r-hGH treatment is difficult, as the short-term burden of injection administration is often more apparent than the long-term benefits of therapy [5]. Daily injections or needle-free administration is required, and treatment must be sustained over a prolonged period. Treatment fatigue may have a negative impact on adherence for patients taking r-hGH medication on a long-term basis, as suggested by the observation that chronicity of disease is a factor that influences adherence to therapy [6]. Studies looking at adherence to r-hGH treatment have been constrained by the problem of recording adherence, but results have shown that non-adherence is a problem in some patients [4-7]. One UK study highlighted how frequent poor adherence with r-hGH therapy can be: more than 1 injection/week was missed by 39% (29/75) of children, whilst 23% (17/75) missed >2 injections/week [5].

The accurate monitoring of adherence rates is important as it enables poor adherence to be detected and acted upon [8]. It can enable the physician to eliminate poor adherence as a reason for sub-optimal growth response and be more confident in their patient management decisions. Innovation in r-hGH delivery devices has sought to improve adherence by simplifying injection administration, making it less painful and more convenient, thereby improving patient acceptability of devices [9-12]. The electronic auto-injector device, easypod™, has a number of features, such as preset dosing, an electronic skin sensor and adjustable injection settings, designed to make daily administration of r-hGH easier, more comfortable and convenient; it also allows accurate monitoring of treatment adherence [13]. easypod™ has an injection log that automatically records injection history. This information can be accessed by patients or downloaded at their clinic to show which injections, if any, have been missed. Although patient opinion of the electronic auto-injector has been previously studied, albeit in a limited number of patients (n = 61) [14,15], adherence to treatment whilst using this device has not been explored at all.

We report here the results from the first study of adherence to treatment as recorded by the auto-injector. The primary objective of this study was to evaluate adherence to r-hGH therapy over 3 months of use. In addition, the survey explored perceptions of the electronic auto-injector device in a large multinational cohort. Study outcomes were also assessed separately for children who were either new to or had experience with r-hGH therapy, in order to identify any differences in adherence to therapy or opinions of the device.

## Methods

### Participants

Eligible children were aged ≤18 years and were either treatment-naïve or already receiving GH treatment, but dissatisfied with their current self-injection device. All children had conditions for which treatment with r-hGH (Saizen^®^, Merck Serono S.A. - Geneva, Switzerland, an affiliate of Merck KGaA, Darmstadt, Germany) was indicated, based on local licensing (GHD, TS, CRF or SGA). Children were excluded if they were currently participating in a therapeutic trial or had done so in the preceding 3-month period. Children were also excluded at any point during the survey if they failed to bring their electronic auto-injector to a consultation.

Recruitment took place in 206 centres, across 15 countries, specializing in the management of growth disorders in children.

### Study design

This was a multicentre, multinational, observational study. Treatment with r-hGH was initiated in accordance with the registered dosage regimen for those new to treatment, or continued as usual for children already receiving this therapy. No treatment changes were made. Similarly, no specific procedures, examinations or follow-up visits were required for the survey. Where applicable, according to local regulations, institutional approvals and appropriate consents were obtained. The guardians of the children consented to the survey.

### Objectives

The primary objective was to assess adherence to r-hGH treatment, following introduction of the electronic auto-injector, comparing adherence patterns of treatment-naïve and treatment-experienced children. The secondary objective was to assess the acceptability of the device.

### Outcome measures

Adherence to treatment was measured using data recorded by the injection device and reported via the survey. For reported adherence, children or their parents completed a questionnaire in which they were asked to tick one of a selection of boxes to indicate how many doses they had missed during each month of the 3-month period, and to give the main reason for missing doses. Recorded adherence data (from the auto-injector) were compared with reported adherence data (from the survey).

Acceptance of the electronic auto-injector device was evaluated using a standardized questionnaire administered by the nurse or investigator after 3 months of use (Table 1). Children or their parents were asked to rate the device (with respect to ease, speed, comfort of use, specific device features and future use) using 5-point scales or multiple-choice responses. The survey was completed by the child or parent based on each family's preference. This information was not collected during the survey; hence no psychometric properties of the survey were made available.

**Table 1:** Survey questions evaluating acceptability of the electronic auto-injector

Question	Response options				
How easy do you find the preparation steps of easypod™?	1 Very easy	2 Easy	3 Average	4 Not easy	5 Not easy at all

How long does it normally take you to prepare your growth hormone injection with easypod™?	1 Very short	2 Short	3 Average	4 Long	5 Very long

How long does it normally take you to inject Saizen using easypod™?	1 Very short	2 Short	3 Average	4 Long	5 Very long

For each injection site, which setting for the needle insertion depth do you prefer?					
Thighs	Low	Medium	High	Not applicable	
Belly	Low	Medium	High	Not applicable	
Buttocks	Low	Medium	High	Not applicable	
Arms	Low	Medium	High	Not applicable	

Which setting for the injection speed do you prefer?	Low	Medium	High		

Did the injection with easypod™ hurt?	Yes	No			

If yes, did the injection hurt as much as you thought it would?	1 Much less	2 Less	3 Average	4 More	5 Much more

Has easypod™ been easy to use?	1 Very easy	2 Easy	3 Average	4 Not easy	5 Not easy at all

Did you feel comfortable using easypod™?	1 Very comfortable	2 Comfortable	3 Average	4 Not comfortable	5 Not comfortable at all

If you had a choice, would you continue to use easypod™?	Yes	No			

Do you think easypod™ is easy to hold/grip?	1 Very easy	2 Easy	3 Average	4 Not easy	5 Not easy at all

Do you think easypod™ is compact/portable?	1 Very compact	2 Compact	3 Average	4 Not compact	5 Not compact at all

Do you think easypod™ has an attractive/nice design?	1 Very attractive	2 Attractive	3 Average	4 Not attractive	5 Not attractive at all

Do you think it is easy to change the cartridge on easypod™?	1 Very easy	2 Easy	3 Average	4 Not easy	5 Not easy at all

Do you think it is easy to change the needle on easypod™?	1 Very easy	2 Easy	3 Average	4 Not easy	5 Not easy at all

Do you think injection using the easypod™ is quiet?	1 Silent	2 Quiet	3 Average	4 Quite noisy	5 Noisy

### Statistical analysis

#### Sample size

The sample size was based on the primary assessment criterion of treatment adherence (the definition of treatment adherence used is given below) and the objective was to demonstrate the equivalence between treatment-naïve and treatment-experienced children in terms of treatment adherence. However, as the type of patient (treatment-naïve or treatment-experienced) recruited could not be controlled for, there was a bias towards a greater proportion of treatment-naïve children. It was therefore decided to perform the statistical analysis in a descriptive way.

#### Data analysis

Adherence was evaluated over the 3-month survey period as a whole and for each month separately to explore short-term changes in adherence. The number of injections prescribed per week was taken into account in the calculation of adherence. Since adherence is expected to be high (80-95% [6]) during the first 3 months of treatment, children were considered adherent to treatment if they received more than 92% of the prescribed doses over a specified period; i.e. a maximum of two daily injections missed per month or six daily injections for the 3-month period.

Three different imputations for missing data were used. First, if the number of injections scheduled per week was not known, the most conservative case scenario (i.e. seven scheduled injections per week) was assumed. Second, when calculating percentage adherence using the 'number of missed injections' questionnaire box selected, the midpoint value for each tick box was imputed, i.e. when the tick box '1-3 injections missed' was ticked, the value '2' was imputed.

Third, for data related to the number of doses missed, the following imputation rule was applied: for respondents answering 'no' to the question "did you miss at least one injection?", but not completing answers for the number of injections missed during each of the three 4-week periods, it was assumed that the first answer was correct (i.e. zero injections were missed).

Furthermore, when information on the number of injections missed per 4-week period was completely missing, imputation analyses were performed using data from completed periods.

Analyses of parents' and children's device acceptance were descriptive. However, for the mean adherence values, 95% confidence intervals were calculated. The following statistical tests were performed: Fisher's exact test was used to compare adherence between treatment-naïve and treatment-experienced children and the Wilcoxon signed rank test was used to compare recorded adherence and reported adherence. The significance level of the tests was set at 5%.

## Results

### Participants

Overall, 834 children using the electronic auto-injector were recruited over a period of 1.5 years. Of these, 824 children were included in the evaluable population, with four children excluded due to incomplete data (age missing [n = 1], status missing [n = 3]) and six children excluded due to being aged >18 years. Of the 824 evaluable children, 601 were treatment-naïve and 223 were treatment-experienced. In total, 682/824 (82.8%) children provided 3 months of data: 496/601 (82.5%) of treatment-naïve children and 186/223 (83.4%) of treatment-experienced children. The remaining children had incomplete information for the numbers of reported and recorded injections.

**Figure 1:**
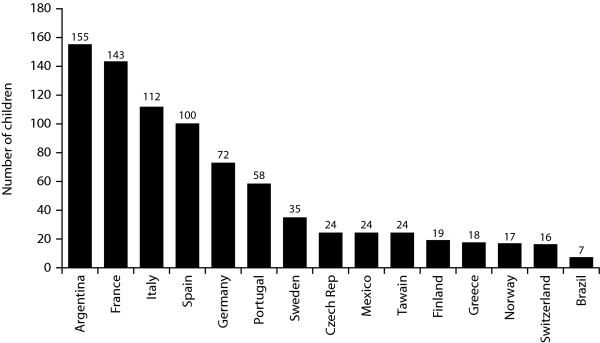
**Geographic distribution of survey participants in the evaluable population**.

The 824 children were recruited from 206 centres across 15 countries. The number of children recruited from each country is shown in Figure 1. Characteristics of the survey population are shown in Table 2. Parents administered the injections for 52.8% (430/815) of children. The median (range) age of children administering their own injections was 13 (5-18) years and for children whose parents took control of injections was 9 (1-18) years. Most children (564/798; 70.7%) were prescribed seven injections per week, with the remaining children prescribed only six injections per week.

**Table 2:** Characteristics of the evaluable survey population

	Total (N = 824)	Treatment-naïve (n = 601)	Treatment-experienced (n = 223)
Boys, n (%)^a^	462 (56.1)	358 (59.6)	104 (46.9)
Age, median (range), years	11 (1-18)	11 (1-18)	11 (2-18)
Indication, n (%)^b^			
GHD	543 (66.4)	407 (68.1)	136 (61.8)
TS	80 (9.8)	46 (7.7)	34 (15.5)
CRF	14 (1.7)	10 (1.7)	4 (1.8)
SGA	125 (15.3)	98 (16.4)	27 (12.3)
Other	56 (6.8)	37 (6.2)	19 (8.6)
Administration of injections^c^			
Child	315 (38.7)	224 (37.5)	91 (41.7)
Parent	430 (52.8)	317 (53.1)	113 (51.8)
Other	7 (0.86)	6 (1.01)	1 (0.46)
Child and parent	59 (7.2)	46 (7.7)	13 (6.0)
Parent and other	4 (0.49)	4 (0.67)	0
Number of scheduled injections per week^d^			
6	234 (29.3)	177 (30.2)	57 (26.9)
7	564 (70.7)	409 (69.8)	155 (73.1)

^a^One child missing from the treatment-experienced group.

^b^Six children missing: three each from the treatment-naïve and treatment-experienced groups.

^C^Nine children missing: four from the treatment-naïve group and five from the treatment-experienced group.

^d^Twenty-six children missing: fifteen from the treatment-naïve group and eleven from the treatment-experienced group.

CRF -- chronic renal failure; GHD -- growth hormone deficiency; SGA -- small for gestational age; TS -- Turner syndrome.

### Recorded adherence

Looking at the recorded dose history for only those children who provided complete data sets, 87.5% of children met the criteria for adherence to treatment over the 3-month period. Adherence was significantly higher in treatment-naïve children (89.7%) than in treatment-experienced children (81.7%; Fisher's exact test FI(X) = 7.577; *p *= 0.0062) (Table 3a). There was little change in the proportion of children reaching the adherence criterion over the 3 months (90.5% in month 1, 87.1% in month 2 and 88.9% in month 3) (Table 3b).

**Table 3:** Adherence to treatment ( > 92% of injections taken)

	Total (N = 824)	Treatment-naïve (n = 601)	Treatment-experienced (n = 223)
**(a)**			
*Recorded adherence - all children*			
Overall, n (%)*	649/772 (84.1)	484/561 (86.3)	165/211 (78.2)
*Recorded adherence - children who provided 3 months of data*			
Overall, n (%)	597/682 (87.5)	445/496 (89.7)	152/186 (81.7)
*Reported adherence - all children*			
Overall, n (%)*	677/790 (85.7)	497/573 (86.7)	180/217 (83.0)
*Reported adherence - children who provided 3 months of data*			
Overall, n (%)	607/673 (90.2)	447/489 (91.4)	160/184 (87.0)

**(b)**			
*Recorded adherence - children who provided 3 months of data*			
Month 1, n (%)	617/682 (90.5)	458/496 (92.3)	159/186 (85.5)
Month 2, n (%)	594/682 (87.1)	441/496 (88.9)	153/186 (82.3)
Month 3, n (%)	606/682 (88.9)	449/496 (90.5)	157/186 (84.4)

(a) Adherence as recorded and reported by children/parents overall over the 3-month survey. (b) Adherence as recorded by easypod™ over each month of the 3-month survey

*Overall adherence calculated by imputation of missing period(s) using non-missing period(s)

Over the 3-month period, there was a slight, non-significant difference in the proportion of children adherent to treatment according to who administered the injections: 84.8% in all children for whom their parents administered the injections versus 82.7% in all children who administered the injections themselves (using imputed data). This trend was driven by adherence data from month 3, as adherence was similar during months 1 and 2.

**Figure 2:**
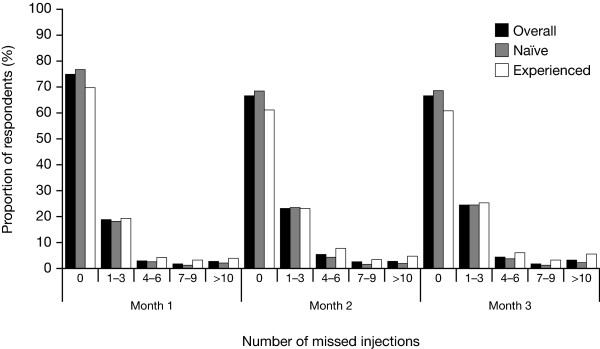
**Missed injections during each month of the survey according to the downloaded dose history (after imputation for missing data)**.

Over the whole 3-month period, 51.4% (397/772) of children were recorded to have missed one or more injections. There was a gradual increase in the number of children missing injections from one month to the next (Figure 2). In month 1, 75.1% (535/712) of children were recorded as missing no injections, in month 2, 66.7% (481/721), and in month 3, 66.7% (480/720). The number of children missing 1-3 injections per month increased over the same period (from 18.5% [132/712] to 24.4% [176/720]). Few children missed >10 injections: only 3.1% (22/720) in month 3.

**Figure 3:**
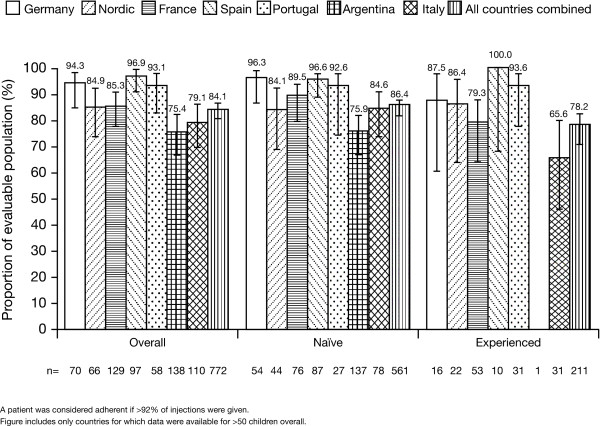
**Recorded adherence according to country (after imputation for missing data using non-missing period(s))**.

The mean recorded adherence was also assessed for individual countries (only in countries for which data were available for >50 children). Of these countries, overall recorded adherence was greatest in Spain (96.9%) and lowest in Argentina (75.4%; Figure 3). Adherence was higher for treatment-naïve children than treatment-experienced children in all countries with the exception of the Nordic countries (Finland, Norway and Sweden), Portugal and Spain, for which adherence was more comparable between the two patient types (Figure 3).

### Reported adherence

The self-reported adherence results indicated that 90.2% of children were adherent to treatment over the 3-month period (Table 3a). Using the self-reported survey data, 51.5% (421/817) of children were reported to have missed ≥1 injection over the 3-month period. The most common reason (given by 43.7% of eligible respondents) for missing a dose was forgetfulness; other reasons (cited by >10% of eligible respondents) included the device not working, running out of cartridges/needles and being away from home (Table 4).

**Table 4:** Most common reasons given for missed injections

	Total (n = 412)	Treatment-naïve (n = 296)	Treatment-experienced (n = 116)
Forgot injection	180 (43.7)	135 (45.6)	45 (38.8)
Device not working	75 (18.2)	54 (18.2)	21 (18.1)
Short vacations, slept away from home	52 (12.6)	31 (10.5)	21 (18.1)
Ran out of cartridges/needles	53 (12.9)	43 (14.5)	10 (8.6)
Forgot drug/easypod™	32 (7.8)	25 (8.5)	7 (6.0)
Sick	35 (8.5)	23 (7.8)	12 (10.3)
Afraid of using easypod™	12 (2.9)	5 (1.7)	7 (6.0)
Tired of injections	18 (4.4)	7 (2.4)	11 (9.5)
Reconstitution issues	13 (3.2)	11 (3.7)	2 (1.7)
Lack of caregiver to provide injection	5 (1.2)	3 (1.0)	2 (1.7)
Too complicated/forgot how to use device	4 (0.97)	2 (0.68)	2 (1.7)
Other	11 (2.7)	6 (2.0)	5 (4.3)

Data values represent number (percentage) of respondents who gave the indicated response as a reason for missing injections

### Recorded versus reported adherence

Comparing recorded against reported adherence showed that more children had higher reported adherence than recorded adherence at each time point as well as overall (90.2% vs 87.5%; Table 3a). An assessment of difference and concordance between reported and recorded adherence revealed that over months 1-3, the recorded and reported adherence values were equal for 84.3% of all children, but in 16% of children, reported adherence values were different to recorded adherence values: reported adherence was higher than recorded adherence for 12.8% of children and recorded adherence was higher than reported for 3.0% (mean percentage difference between recorded and reported adherence -0.85; *p *< 0.0001 using the signed rank test). Similar trends were observed in treatment-naïve and treatment-experienced children. A similar number of questionnaires were being administered prospectively and retrospectively; however, an exploratory analysis showed no differences in adherence between the two approaches were observed.

### Acceptability of the electronic auto-injector device

#### Device preparation

Overall, 82.5% (664/805) of participants found the electronic auto-injector easy/very easy to prepare. Only 2.2% (18/805) of respondents rated the injection preparation as not easy at all (Table 5). The majority (684/806, 84.9%) of respondents also rated the duration of injection preparation as short/very short (Table 5).

**Table 5:** Acceptance of the electronic auto-injector

		Number of respondents	Most positive answer	→			Least positive answer
			
			1	2	3	4	5
Device preparation	Ease	805	53.5	28.9	12.6	2.7	2.2
	Duration	806	53.2	31.6	12.7	0.99	1.5
Device use	Ease	779	62.6	29.8	5.8	1.0	0.77
	Duration	805	53.0	31.9	13.3	1.2	0.50
	Comfort	804	57.5	31.6	7.6	1.7	1.6
Device features	Grip	813	59.7	28.2	9.0	2.1	1.1
	Size	803	52.4	26.0	15.6	3.6	2.4
	Attractiveness	807	56.6	28.3	12.8	1.5	0.87
	Ease of cartridge change	801	58.8	27.0	10.4	2.9	1.0
	Ease of needle change	809	56.9	26.1	12.2	3.7	1.1
	Noise	807	51.7	32.3	12.1	2.6	1.2

Figures indicate the percentage of survey participants giving the indicated rating (scale of 1-5) in response to specific questions about the electronic auto-injector.

#### Device use

When questioned about the ease and duration of injection, 92.4% (720/779) of respondents said that it was easy/very easy to use and 85.0% (684/805) rated the duration of injection as short/very short (Table 5).

Of the possible choices for injection location (thighs, belly, buttocks or arms), the thighs were the preferred site (88.3% [686/777] of children injected in the thighs, whereas other sites were used by less than 53.8%). Overall, the default setting (medium) was preferred for both injection depth (72.3% [562/777] when injecting in the thighs; 35.7% [231/647] when injecting in the belly; 41.9% [279/666] when injecting in the buttocks; and 43.5% [285/656] when injecting in the arms) and injection speed (71.1% [566/796] when injecting in any location).

The device was described by 89.1% (716/804) of participants as comfortable/very comfortable to use (Table 5). The majority of respondents (61.5% [480/780]) reported experiencing no pain when injecting with the electronic auto-injector. Those patients who experienced pain were then asked whether the injection hurt as much as they were expecting (although this question was also answered by some of those who said that they did not experience pain). Over half (57.9% [210/363]) of respondents to this question described it as being less or much less painful than expected. Few respondents (13.5% [49/363]) reported that the injection hurt more/much more than they thought it would.

#### Device features

Survey participants were also asked to answer questions concerning specific features of the electronic auto-injector. In total, 85.8% (687/801) and 82.9% (671/809) stated that the cartridge and needle, respectively, were easy/very easy to change (Table 5). Most participants found the device easy to hold and compact, as well as attractive and silent/quiet on injection (Table 5).

#### Future use of the device

At the end of the 3-month survey period, 91.8% (732/797) of respondents stated a desire to continue using the electronic auto-injector: 92.7% (542/585) of treatment-naïve and 89.6% (190/212) of treatment-experienced children.

For children who did not want to continue using the device, 74.6% (44/59) were recorded as being adherent to treatment.

## Discussion

This multinational, observational survey of over 800 children using an electronic auto-injector device for up to 3 months found a good level of adherence to therapy. The dose history that had been automatically recorded by the device and reviewed by the physician showed that 87.5% of children were adherent to therapy (injecting at least 92% of doses over the 3-month period). Furthermore, 75.1% of all children did not miss any injections in month 1, 66.7% in both months 2 and 3.

Adherence to treatment has an important impact on overall treatment outcome. In a group of poorly compliant patients, growth rates were found to be significantly lower compared with patients who missed fewer doses [4]: height velocity in patients missing >15 injections per month (equivalent to >3-4 per week) was 6.3 cm/year compared with 9.4 cm/year in those missing 11-15 doses per month (*p *< 0.03). It has also been estimated that growth velocity suffers a significant decline if >2 injections per week are missed [5]. In this survey, it was possible to monitor how many injections were missed during each month of the survey by looking at the dose-history data from the electronic auto-injector. By scaling up to find a comparable figure for the proportion of patients who missed >2 injections per week, only a relatively small percentage of children had a level of non-adherence that would have a serious impact on growth ( < 5% of children missed ≥7 injections in month 3).

Adherence was higher in treatment-naïve children compared with treatment-experienced children (89.7% vs 81.7%) over the course of the survey. This observation suggests that a drop in adherence may occur with increasing duration of treatment: longer-term users may be less enthusiastic or motivated about adhering to treatment compared with those users new to treatment, who may be more diligent. This agrees with previous studies with GH [5] and other therapies taken on a long-term basis [16,17]. Even within the short duration of the survey, a moderate decline in adherence rates was observed. The very good levels of recorded adherence during the first month, when 75.1% of children missed no injections, fell in the second and third months, with 66.7% of children missing no injections at month 3. As this survey only followed children for 3 months, an evaluation of adherence over a longer time period is currently planned.

Self-reported adherence, collected via the survey, showed slightly higher rates of adherence than the dose-history data (90.2% vs 87.5%). Although asking the user about their injection habits may be the most straightforward approach to evaluating adherence, these results show that it may not always be a reliable and accurate method. Missed doses may be forgotten and adherence overestimated. Electronic recording of the dosing history using a device with an integrated dose log offers an improved, objective method of accurately monitoring adherence.

The rates of compliance reported here compare well with previous studies [4,5]. Data from an American GH registry based on physician-reported adherence, indicate that 76-85% missed 0-3 doses per month, over a 24-month period [4]. In the current survey, a child was considered adherent to treatment if they missed a maximum of two injections per month. Therefore, a proportion of the children considered adherent in the American GH registry (those who missed three injections per month) will not have been considered adherent in our study. In a study of 75 children attending a UK clinic, an assessment of adherence was based on prescriptions over 12 months [5]. The frequency of missed injections was estimated to be 0 in 36% of children, up to 1 per week in 25% of children, >1-2 per week in 16% of children and >2 per week in 23% of children.

Although the factors influencing adherence and persistence in GH treatment were not explored here, misperceptions about the consequences of missed GH doses, discomfort with injections, dissatisfaction with treatment results and inadequate contact with healthcare providers are among the key reasons reported in other studies [6,7]. Coaching by healthcare professionals and ongoing discussions about the benefits of treatment are important to achieve good adherence [18]. Better patient autonomy in managing their own condition is also predictive of treatment adherence, as is involving the patient in treatment decisions [19]. Patients may be more likely to be adherent to treatment if they are allowed to choose the delivery device [5,20]. Potential barriers to better adherence include the use of complicated delivery devices [18], suggesting that having a good perception of the delivery device is a factor that may also influence adherence.

The current survey also showed that children and their parents had a very good perception of the electronic auto-injector after 3 months of use, rating specific features of the device highly. The majority considered the device to be quick, easy and comfortable to use. There was little difference between treatment-naïve and treatment-experienced children in their responses to questions.

These results support the findings of previous, smaller (up to 61 patients), shorter-term (up to 60 days) studies, which together indicate a high level of patient acceptance of the electronic auto-injector for daily administration of r-hGH [14,15]. Results from an open-label multicentre survey that included 61 patients showed that the majority of patients had a good overall impression of the device after 60 days of use [14]. In addition, the nurses/physicians who trained them how to use the device also rated the device favourably with respect to participants' ease in learning to use the device [14]. Unlike previous user trials of the electronic auto-injector [14,15], the current study is the first to include data on adherence as captured using the device.

In a survey conducted at a UK hospital, patients commencing GH therapy were given the freedom to choose their delivery device [20]. Of those switching devices, 74% changed to the electronic auto-injector. None of the patients who started on this novel device later switched to using another device. Although the present survey did not provide children or their parents with a free choice of injection device, at the end of the survey, 92% of children/parents stated that they would like to continue using it. Interestingly, adherence in children who did not want to continue using the device was slightly lower than that in the overall population (when considering all children in the evaluable population, 74.6% vs 84.1%, respectively).

## Conclusions

This survey of 824 children showed a high level of compliance to daily r-hGH administration using easypod™ over a 3-month period, with almost 90% of children meeting the criterion for adherence to treatment. A significantly higher adherence rate was observed in treatment-naïve children compared with treatment-experienced children. In addition, children/parents liked the device and found it easy to use for daily administration of r-hGH even if they did not have any previous experience of injecting. Moreover, almost all wanted to continue using it. Such acceptance of devices for r-hGH administration should reduce the burden of daily treatment and may improve adherence to therapy. Good adherence, as demonstrated here, should lead to optimal efficacy and allow children to reach their height potential.

## Competing interests

MB, MC and MH-S declare that they have no competing interests. SL and MZ are employees of Merck Serono S.A. - Geneva, Switzerland, an affiliate of Merck KGaA, Darmstadt, Germany.

## Authors' contributions

MB: Critically reviewed and revised the manuscript. MC: Critically reviewed the manuscript. MH-S: Critically reviewed the manuscript and has been a principal investigator in Sweden. SL: Was involved in the statistical design and analysis of the study and critically reviewed the manuscript. MZ: Was involved with the design and analysis of the study and critically reviewed the manuscript. All authors have read and approved the final version of the manuscript.

## Pre-publication history

The pre-publication history for this paper can be accessed here:

http://www.biomedcentral.com/1472-6823/11/4/prepub
